# Unhealthy habits persist: The ongoing presence of modifiable risk factors for disease in women

**DOI:** 10.1371/journal.pone.0173603

**Published:** 2017-04-12

**Authors:** Cassandra Szoeke, Christa Dang, Philippe Lehert, Martha Hickey, Meg E. Morris, Lorraine Dennerstein, Stephen Campbell

**Affiliations:** 1 Faculty of Medicine Dentistry and Health Sciences, University of Melbourne, Melbourne, Australia; 2 Institute for Health and Ageing, Australian Catholic University, Melbourne, Australia; 3 Department of Mathematics and Statistics, University of Mons, Mons, Belgium; 4 School of Allied Health, La Trobe University, Melbourne, Australia; 5 Melbourne Health, Melbourne, Australia; Fondazione Toscana Gabriele Monasterio, ITALY

## Abstract

**Objectives:**

Vascular disease remains a leading cause of death. There are several vascular risk factors identified that can mitigate development of disease in ageing. We examine reported rates of modifiable risk factors in women responding to an online health questionnaire advertised by popular media.

**Methods:**

A sample of 26 620 women aged over 18 was examined in 2015 with a cross-sectional health questionnaire. The questionnaire included self-reported health, mood, lifestyle and vascular risk factors.

**Results:**

There remains high rates of modifiable risk factors present in women. The vast majority of women (80%) reported not eating enough fruit and vegetables. Compared to the guidelines for health, the majority did not perform enough weekly physical activity (70%) and more than half the participants were overweight (54%). Sufficient fruit, vegetables, fish, legumes and physical activity were reported in less than 30% of women!

**Conclusions:**

Women continue to report low rates of physical activity, fruit and vegetable intake and higher BMI than recommended for good health, despite worldwide health promotion activities aimed at changing these lifestyle factors. Programs to support healthy living need to be reviewed and revised to reduce the burden of vascular disease and dementia in women. Previous guidelines are not having the important impact they should, particularly in women.

## Introduction

The leading cause of death in women is vascular disease [1]. This includes cardiovascular, cerebrovascular and peripheral vascular disease and has been well known for over 20 years [2]. The largest burdens of disability in Australian women are vascular disease and dementia; in 2011, 13.7% of female deaths were caused by coronary heart disease, 9.5% by cerebrovascular diseases, and 9.2% by dementia and Alzheimer’s disease [3]. There are several known modifiable risk factors which, if targeted, can reduce the burden of vascular disease [4–7], with evidence mounting that reduction of these same risk factors can halve the cases of dementia [8]. Public health programs have demonstrated that a modification of risk factors was responsible for a dramatic decline in death and disease in the late 1960s and 1970s [9]. The population-based approach to prevention has been globally successful [10–13] via risk factor reduction [14]. This has been particularly the case for men, as shown in previous trials [5, 15].

It is concerning that improvements in lifestyle and associated health outcomes have not been evenly distributed between the sexes. Since the late 1980s, more women than men have suffered vascular disease [16, 17]. Recent reports also show that women continue to have more vascular disease than men [18] and make up more than half of all deaths from cardiovascular disease [19]. Women are currently more likely to suffer stroke (cerebrovascular disease)–for every 64 male deaths due to stroke, there are 100 female deaths [20]. Age-matched women are more likely than men to develop cognitive impairment and dementia [21].

**Fig 1:**
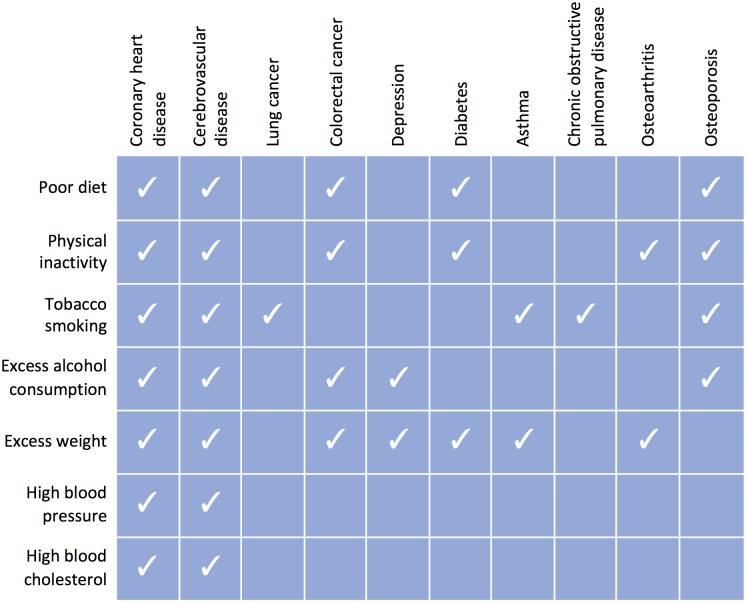
The contributions of modifiable risk to common chronic diseases. Source: Institute of Health and Welfare 2002. Chronic diseases and associated risk factors in Australia, 2001. Canberra: AIHW.

The influence of modifiable risk factors on death and disease is also significantly different between men and women, and there are established examples in heart disease where risk factors and approaches to prevention are entirely different [22]. It is important to better understand the prevalence of risk factors in women. Women over 45 with two or more risk factors have a 31% chance of having a major cardiovascular event by the age of 80 compared to women with no risk factors, who only have a 4% risk [18]. Hypertension, smoking, high cholesterol, low fruit and vegetable intake, overweight, lack of physical activity and alcohol are responsible for the attributable deaths in high income countries [23]. These risk factors contribute to a number of diseases (Fig 1) and are known to cluster together in adults [24, 25].

### Objectives

In this paper, we report the proportion of Australian women reporting health risks and behaviours from a recent national electronic health questionnaire. We compare reported health behaviour to the recommendations provided by the Institute of Health and Welfare to cease smoking, reduce alcohol consumption, maintain a healthy weight, increase exercise and eat at least 5 serves of fruit and vegetables daily (Table 1). We report the compliance with available health recommendations.

**Table 1:** Summary of the Institute of Health and Welfare recommendations on lifestyle risk factors to prevent disease.

Modifiable risk factor	National recommendations	Reported health behaviour
Body Mass Index	BMI < 25 [1]	Calculated from self-reported height and weight
Exercise	> 150 minutes moderate exercise each week [2]	How many minutes of moderate activity or exercise do you do per week (involves effort, but you can still talk)? None < 75 minutes 75–149 minutes 150–210 minutes 210 + minutes
Nutrition	5 serves of vegetables and 2 serves of fruit a day [3]	How many fruit and vegetable portions do you have per day? None 1–2 3–4 5 or more
Alcohol	No more than 2 standard drinks per day [4]	How many alcoholic drinks do you have per week? (1 drink = 1 pot or 150ml wine) None 1–7 (1 drink/day) 8–14 (1–2 drinks/day) 15–28 (2–4 drinks/day) 29+ (4+ drinks/day)

^1^. Institute of Health and Welfare, Australia's Health 2012: In brief. 2012: AIHW.

^2^. DOHA, Physical Activity Guidelines. Department of Health and Ageing, 2010.

^3^. National Health and Medical Research Council, Australian Dietary Guidelines, Department of Health and Ageing, Editor. 2013, National Health and Medical Research Council.

^4^. National Health and Medical Research Council, Australian guidelines to reduce health risks from drinking alcohol. 2009, Commonwealth of Australia.

## Methods

A new online health survey was made freely available to all Australians on 9^th^ October 2014. The survey had not been validated prior to implementation. All participants were self-selected to complete the health questionnaire, and gave written consent to the use of their aggregate, non-personal information. The de-identified data from all adult female participants who had participated in the health questionnaire up to 21^st^ January 2015 was audited. This report describes the data on all women over the age of 18 who had registered over 3 months on the online platform from 9^th^ October 2014 to 21^st^ January 2015. Ethics approval for the study was granted to the Women’s Healthy Ageing Project by the University of Melbourne Human Ethics Committee to examine this initial enrolment data provided by the administrators of the survey (approval number 1544129.1).

All questions were grouped into categories representing known modifiable risk factors, and participants were determined to be at risk for each category if their aggregate responses were outside of national health recommendations. Total number of health risks were then calculated. A subset of the questionnaire formed the key variables of interest in this analysis, which are shown in Table 2. All data were recorded categorically except for height and weight, which was used to calculate body mass index (BMI) using the equation BMI = kg/m^2^. The response categories were selected to readily match with national guidelines with no need for re-categorization. Statistical analyses were performed using SPSS for Windows 11.1. Descriptive statistics were used to report the proportion of participants within various categories of lifestyle. Odds ratios were calculated for each pair of risk factors, and significance tests were used to examine the relationships between variables of interest. All significance tests were adjusted for age.

**Table 2:** Key variables of interest.

Variables	Responses collected
**Sociodemographic:**	
Age	Month, year of birth
Employment status	Employed Self-employed Volunteer work Retired Not employed
**Lifestyle:**	
Cigarette consumption	Current smoker Past smoker–quit more than 3 months ago Past smoker–quit less than 3 months ago
Physical activity	Number of minutes per week engaged in moderate and vigorous (unable to talk during) physical activity Number of steps recorded by electronic device per day (if applicable)
Alcohol consumption	Number of standard drinks per week (and average number per day) Spread of consumption throughout the week
Fruit and vegetable consumption	Number of serves per day
Red or processed meat meals	Number of meals per week
Fish or legume meals	Number of meals per week
Fat-fried meals	Number of meals per week
Regular breakfast consumption	Number of substantial breakfasts per week
Sleep	Average hours of sleep per night
Current mental/physical health	Self-rated health
Stress and coping	Self-rated stress and coping
**Vascular risk factors:**	
BMI	Calculated from height and weight provided by respondent and categorised: Underweight (<18.5) Normal (18.5–25) Overweight (25–30) Obese (30–40) Extremely obese (>40)
Systolic blood pressure (SBP)	Normal (<120) Normal-high (120–139) High (140–159) Very high (>160)
Diabetes	Ever diagnosed with diabetes: yes/no

## Results

Of the 26 620 registrants, 97.5% had completed all data in the initial online health questionnaire at the time of analysis. Characteristics of the sample are depicted in Table 3. The majority (70.9%) of participants resided in New South Wales, Queensland and Victoria. Participants were aged 40 on average (SD = 15), ranging from 18 to 100 years of age. Age categories show that there were relatively equal proportions of 18–24; 25–34; 35–44; 45–54 and 55–64 age groups ranging from 18–22% in each category, but there were fewer participants in the 55–64 age group (14.4%) and many fewer in the over 65 age group (5.7%). Of all respondents, 64% were employed and 21% were not employed, with 7% self-employed and 6.3% retired or doing volunteer work.

**Table 3:** Characteristics of the women who completed the online health survey.

Category	Percentage	N
Sociodemographics		
Age 18–24	20.1%	5354
Age 25–34	22.5%	5981
Age 35–44	17.6%	4676
Age 45–54	19.8%	5280
Age 55–64	14.4%	3822
Age over 65	5.7%	1507
Employed	64.0%	17033
Unemployed	21.0%	5585
Self employed	7.0%	1869
Volunteer work	3.0%	804
Retired	3.3%	882
Risk Factors		
Smoker (current or quit in last 3 months)	12.1%	3231
Alcohol consumption (>2 standard drinks per day)	4.5%	1204
Diabetes	3.3%	869
High or very high systolic blood pressure (>140)	3.1%	835

**Fig 2:**
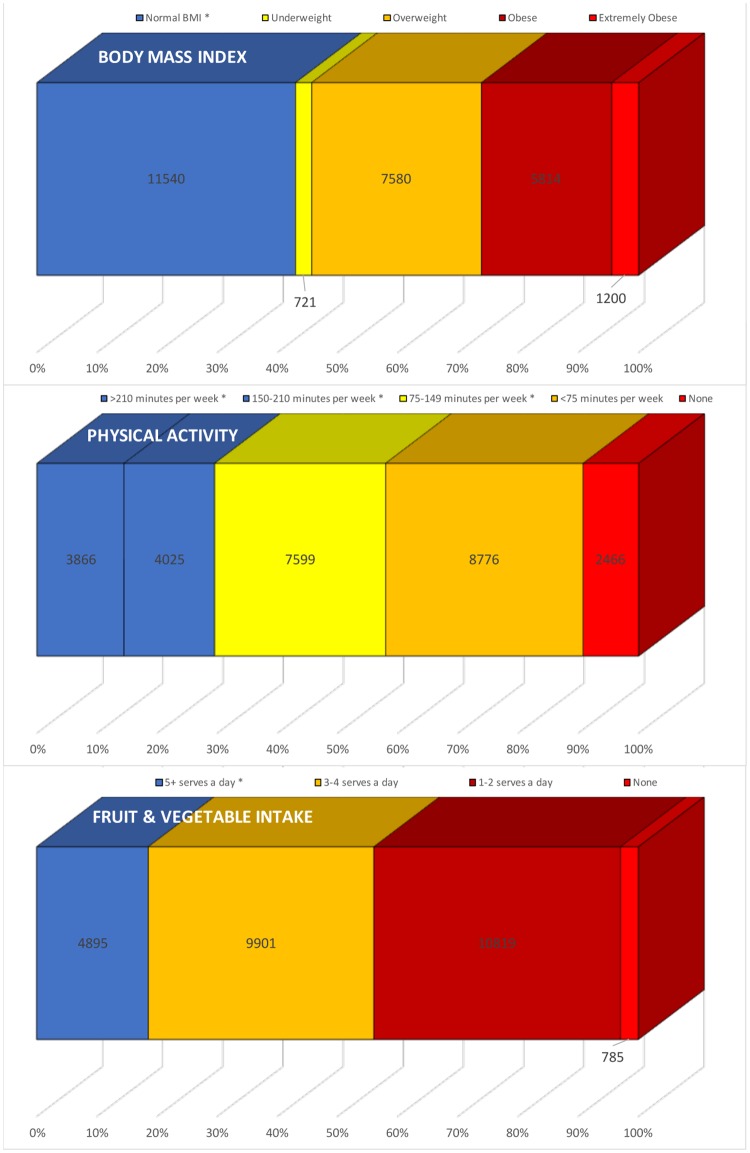
Distribution of lifestyle behaviours in women shown by published category of risk for disease.

65.7% of women reported never smoking with 20.8% (5544) being past smokers and 12.1% smoking or having recently quit. 3.3% of women had diabetes (869) and 3.1% (835) reported high systolic blood pressure. 42.7% (11 364) recorded a normal BMI with 54.4% of participants recording unhealthy weights. Of these, 28.2% (7501) were overweight (BMI 25–30), 21.7% (5776) were obese (BMI 30–35) and 4.5% (1190) were extremely obese (BMI>35) (Fig 2).

Nutritional guidelines had low compliance with 80.3% of women having low fruit and vegetable intake, 26.1% (6933) only had breakfast occasionally, and half of these respondents reporting they never had breakfast. Red or processed meat consumption more than 3 times a week was reported by 41.9% (11 166). Fish or legume meals, currently recommended 3 or more times per week, were occurring in 15.2% of women. Fat-fried meals were reportedly consumed less than once per week by 63.1% (16 808), 28.9% (7680) having these 1–2 times a week and only 6.7% (1775) having these more than 3 times a week (Table 4).

**Table 4:** Reported lifestyle behaviours by category.

Modifiable lifestyle risk factors		
Red or Processed Meat Meals	Percentage	N
5+ times per week	6.9%	(1844)
3–4 times per week *	35.0%	(9322)
1–2 times a week	40.5%	(10 792)
Less than once per week	9.6%	(2546)
None	6.6%	(1761)
Fish or Legume Meals		
5+ times per week	2.8%	(745)
3–4 times per week	12.4%	(3291)
1–3 times a week *	47.0%	(12 523)
Less than once per week	26.8%	(7133)
None	9.7%	(2574)
Fat-fried Meals		
5+ times per week	0.9%	(236)
3–4 times per week	5.8%	(1539)
1–2 times a week	28.9%	(7680)
Less than once per week*	40.1%	(10 678)
None	23.0%	(6130)
Breakfast		
Always	52.6%	(14 011)
4–5 times per week	20.0%	(5326)
2–3 times per week	13.4%	(3564)
Never	12.7%	(3369)

* indicates published recommendations for optimal health.

Only 23% of women noted that they did more than 75 minutes of “activity which made [them] breathe hard” per week. 29.5% did 150 minutes or more of moderate activity per week, who were part of a total of 56.6% doing more than 75 minutes a week. Only 9.2% recorded absolutely no activity at all in the week. Of those who recorded their step count, 8.6% recorded less than 3000 steps; 31.2% recorded 3–6000 steps per day, 40.2% recorded 6–10 000 steps per day and 19.9% recorded more than 10 000 steps per day (Fig 2).

37.9% of respondents (10 087) reported drinking up to a standard drink a day or less than 7 per week, while 48.1% (12808) report not drinking at all. 8.1% (2164) had 1–2 drinks a day or up to 14 per week; 3.4% (910) had 2–4 drinks per day or up to 28 per week; 1.1% (294) had 4 or more drinks a day or over 29 per week. 24.9% of women drank more on weekends, with 6.6% reporting all of their alcohol consumption was on the weekend.

**Fig 3:**
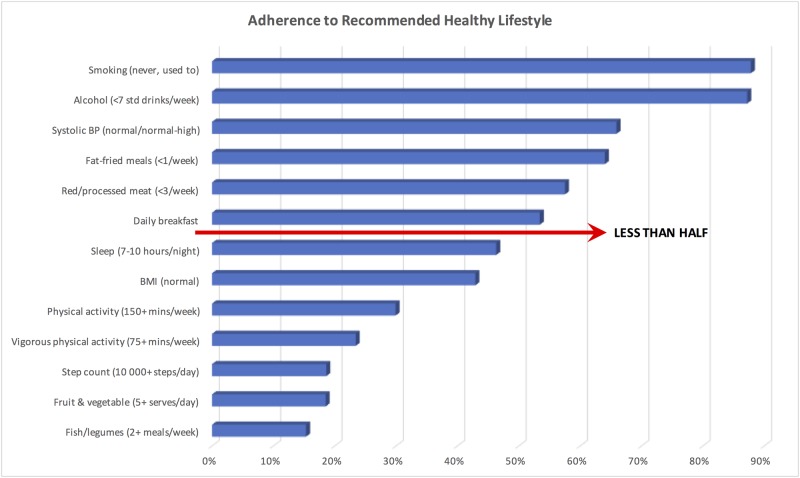
Rate of compliance with recommended health guidelines.

About half (50.6%) of women reported they felt they had good–excellent mental and physical health, 34.2% reported average health and only 13.5% reported that their health was below average. Fig 3 summarises adherence to health recommendations in the community sample, with those recommendations being followed by less than half the participants highlighted.

The total health risk results were not significantly different when including all participants or only including respondents who did not answer “not sure” for any items; therefore, the results presented encompass the entire database. 3.8% of participants had 0 health risks and 18.8% had 1 health risk or less. 60.5% of participants had 3 or more total health risks. Total number of health risks was inversely related to self-rated health; the lower the rating, the greater the number of risks. There was no difference in total health risk by employment status or Australian state of residence.

Individuals who reported not coping well with the pressures of home or work life had significantly more health risks, F(4, 26165) = 659.58, p<0.0005, partial η^2^ = 0.092 and F(5, 20579) = 276.03, p<0.0005, partial η^2^ = 0.06 respectively. All pairwise comparisons were significant at p<0.05. There is a cumulative effect of poor coping at both home and work, where the number of health risks is significantly greater for those who report poor coping in both areas compared to just one, F(14, 20556) = 17.07, p<0.0005, partial η^2^ = 0.01. Additionally, those who reported receiving more help from friends and family had less health risks, F(4, 26120) = 196.86, p<0.0005, partial η^2^ = 0.03. The same effect was found with those who put more effort into maintaining personal relationships.

**Fig 4:**
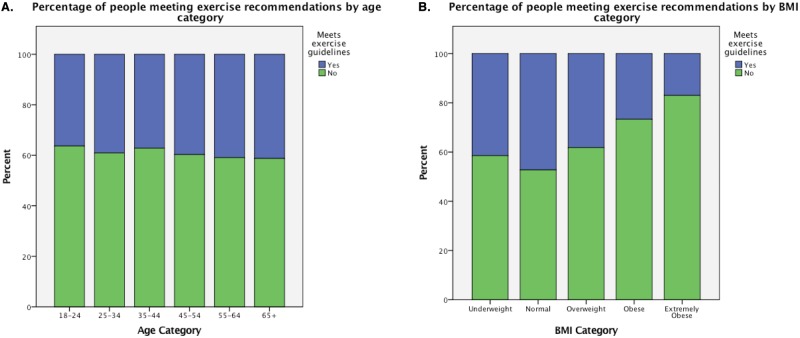
Percentage of people meeting exercise recommendations by age and BMI category.

Examining adherence to physical activity guidelines by BMI category shows that 41.5% of underweight, 47.3% of normal, 38.2% of overweight, 26.7% of obese and 17.0% of extremely obese women met physical activity guidelines. There is a statistically significant difference in exercise risk between these groups, χ^2^(4) = 929.35, p<0.0005, with those who are overweight or obese more likely to be physically inactive. When grouped by age category, 36.3% between 18–24, 39.1% between 25–34, 37.1% between 35–44, 39.7% between 45–54, 40.9% between 55–64 and 41.2% over 65 met exercise recommendations. The difference between age groups is significant, χ^2^(5) = 32.34, p<0.0005 although the association is very small, Cramer’s V = 0.035. See Fig 4.

**Fig 5:**
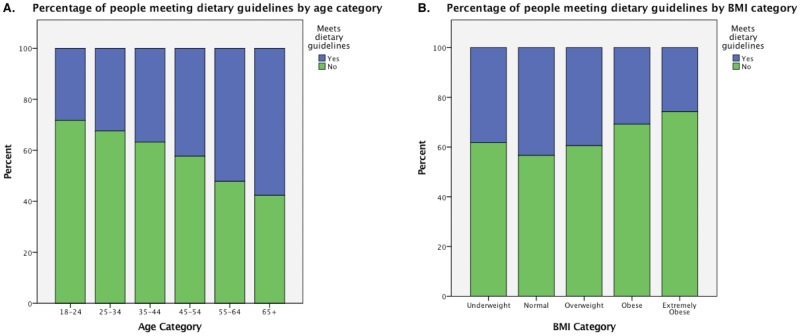
Percentage of people meeting dietary recommendations by age and BMI category.

Compliance with nutrition guidelines was assessed by both BMI and age category. 38.2% of underweight, 43.4% of normal, 39.5% of overweight, 30.8% of obese and 25.8% of extremely obese women followed national dietary recommendations. These differences across BMI category are significant, χ^2^(4) = 339.80, p<0.0005. Additionally, 28.2% of women aged 18–24, 32.4% of women aged 25–34, 36.8% of women aged 35–44, 42.3% of women aged 45–54, 52.2% of women aged 55–64 and 57.6% of women over 65 years of age met the nutrition guidelines. These differences across age category are significant, χ^2^(5) = 893.74, p<0.0005, showing that older women are less likely to be at dietary risk. See Fig 5.

Not meeting the national guidelines for some risk factors was significantly related with not meeting the national guidelines for other risk factors. Odds ratios for the 3 main risk factors, BMI, nutrition and physical activity are presented in Table 5 (see S1 Table for the full set of odds ratios). The majority of the odds ratios were significant at p<0.05. The results show that some unhealthy habits are more likely to result in other unhealthy habits, and likewise with healthy habits. For example, women who followed dietary guidelines were 2.6 times more likely to meet exercise recommendations than those who did not eat well.

**Table 5:** Odds ratios–Likelihoods of not meeting different national guidelines based on risk category for other factors.

	**BMI**	**Nutrition**	**Exercise**
**BMI**		1.4 (1.34–1.48)	1.9 (1.80–1.99)
**Nutrition**	1.4 (1.34–1.48)		2.6 (2.51–2.78)
**Exercise**	1.9 (1.80–1.99)	2.6 (2.51–2.78)	
**Blood Pressure**	3.0 (2.52–3.53)	1.3 (1.13–1.51)	1.8 (1.55–2.11)
**Cholesterol**	1.6 (1.48–1.78)	1.5 (1.35–1.61)	1.4 (1.26–1.51)
**Glucose**	4.4 (3.67–5.38)	1.4 (1.21–1.60)	2.1 (1.82–2.48)
**Smoking**	1.1 (1.03–1.20)	2.0 (1.87–2.21)	1.5 (1.37–1.61)
**Alcohol**	0.7 (0.69–0.81)	1.0 NS (0.89–1.04)	0.8 (0.70–0.81)
**Sleep**	1.3 (1.21–1.34)	1.6 (1.52–1.70)	1.3 (1.24–1.38)
**Stress**	1.3 (1.20–1.34)	1.8 (1.67–1.99)	1.6 (1.51–1.79)

Data are presented as: odds ratio (95% confidence interval). Non-significance is denoted by “NS”. Cells containing increased likelihoods are shaded. The odds ratios represent the likelihood of not meeting national guidelines for row elements if the guidelines for column elements are also not met. For example, the first filled cell indicates that women who did not meet nutrition recommendations were 1.4 times more likely to also not meet BMI recommendations.

## Discussion

This audit of a national convenience sampled database of women showed that, despite implementing public health promotion programs over decades, there remains high rates of modifiable risk factors present in women. The vast majority of women (80%) reported not eating enough fruit and vegetables, although older women were more likely to eat the recommended amount. The majority (70%) did not perform enough weekly physical activity, and more than half (54%) the participants were overweight or obese. Sufficient fruit, vegetables, fish, legumes and physical activity were reported in less than 30% of women. Overall, nearly 40% of women had more than 3 risk factors. Women who reported not coping well with home or work stressors had more health risks, supporting previous research that psychological stress is detrimental to health and wellbeing and can increase the likelihood of unhealthy behaviours [26, 27]. However, greater amounts of social support were associated with less risk factors, suggesting possible protective effects [28]. Respondents who self-rated their physical and mental health as being “poor” exhibited a greater number of risk factors, indicating that the presence of unhealthy habits may not be due to lack of awareness of healthy lifestyle recommendations. It must be noted, however, that such a relationship between self-rated health and unhealthy behaviours is bi-directional and therefore no causal statements can be made. This report is in agreement with past findings that the presence of health risk factors can influence the presence of other risk factors [24, 25]. The converse is also true, that healthy behaviours beget other healthy behaviours; therefore, interventions designed to take advantage of this “cascade” of healthy behaviours may be more beneficial to the population. Other research with Australian women show that higher levels of self-efficacy are associated with greater likelihood of positive changes in exercise and dietary behaviour [29, 30]. Our results indicate that while all women would benefit from increasing physical activity and improved nutrition, certain subgroups of the female population would benefit from targeted programs. More exercise and better diet would be recommended for overweight women, which may also have the effect of reducing BMI, while improving fruit and vegetable intake would be most important for younger women.

As this database was volunteer-based and therefore not random, selection bias could potentially over-represent those with risk factors who are more likely to be interested in a wellness website or, conversely, under-represent risk factors with unhealthy people or those not interested in their health less likely to take part. While it is possible that online methods of administration can change reporting behaviours compared to traditional non-electronic methods [31], previous epidemiological national health surveys showed comparable rates to those above for high BMI (59.8% [32, 33] and 62.8% [34]), inadequate fruit and vegetable intake (72–93% [35]) and insufficient physical activity (60% [36]), suggesting that this database is indeed representative of the population. Similar research in the United States reported that <10% of individuals adhere to national recommendations for all 3 of the aforementioned risk factors [37]; the persistence of such unhealthy habits despite epidemiological evidence of their impact on future health must be addressed.

Vascular risk factors are known to represent the leading cause of death and disability in ageing women. A large body of research shows that many such deaths can be prevented [38]. Despite this, our audit shows that women report high rates of modifiable risk, which, if addressed, could prevent disease. The UK Department of Health estimated that a vascular health check program could prevent at least 9500 heart attacks and strokes a year (2000 of which would be fatal), prevent 4000 people a year from developing diabetes, and detect at least 25 000 people with diabetes or kidney disease a year earlier [39]. Programs improving awareness and prioritising research in the USA have led to a reduction in female deaths from vascular disease [40, 41]. The implementation of such programs in Australia would be widely beneficial.

Medications to treat hypertension, hypercholesterolaemia and recurrent vascular disease have shown great utility in survival, but these studies were predominantly in men. Later work has questioned any benefit of statins in women [42] and even the benefits of aspirin, appear to only be relevant in women over the age of 65 [43]. It is important to note that, since these early studies, there have been landmark policy changes in the USA to ensure research on medical therapies is also conducted in women. A gender disparity still exists in cardiovascular disease burden and outcome, and further gender-specific research is required to determine the most effective methods of prevention and treatment for women [44–46]. Greater understanding of the differential effects of risk factors on women will inform further public health initiatives to increase adherence to national recommendations for healthy living.

Prioritising healthy lifestyles may also be impacted by the evidence that many women remain unaware of the prevalence of vascular disease in women and the importance of modifiable risk factors on their health [47]. Whilst public health campaigns for vascular disease in men have generated high levels of awareness in Australia, only 3–30% of people surveyed were aware that vascular disease was a leading cause of death in women [48, 49]. There is also evidence that the low rates of awareness are not confined to the community, with a lack of physician awareness influencing the management of women with vascular risk factors [50]. Such gender research shows there are specific barriers to prioritising healthy lifestyle in women [49]. The portrayal of exercise and weight loss in the popular media as aesthetic endeavours for women rather than as health promotion activities may raise additional barriers to lifestyle changes; further research should explore the effects of social messages on female health behaviours. These differences in roles and priorities are important to consider when developing guidelines that are relevant to women.

Understanding population health is fundamental to addressing the gaps in evidence and its translation to public health outcomes. The most recent Institute of Health and Welfare survey made contact with 48 579 in-scope households, of which 23 855 questionnaires were categorised as being complete and useable. This represented a response rate for the 2013 survey of 49.1%, which was comparable with the 2010 survey rate of 50.6%. Whilst the internet presents an opportunity for a broad coverage of real-time data collection, the selection bias in convenience sampling needs to be considered. The rates of diabetes and high blood pressure reported in this cohort were slightly below the census data, suggesting that our cohort may be healthier than the general population although about 30% of respondents were unsure of their blood pressure levels. Additionally, some questions in the questionnaire are not easily answered by the average person, such as cholesterol or glucose level; approximately half of all respondents selected “not sure” for these questions, which were included sparingly in analyses for this reason. Self-report of anthropometric measurements such as height and weight may be more approximate than accurate, and this was addressed by grouping respondents into BMI categories rather than treating BMI as a continuous variable. Making health questionnaires available in medical practices may increase definitive and accurate responses, and increase response rate in the older population. The low proportion of participants over 65 is another limitation of the internet-based health survey with the last census data in 2013 reporting those over 65 comprised 14% of the population [51], indicating we missed half of this demographic. This questionnaire did not collect education or socioeconomic information, which would be necessary to further analyse health behaviours with sociodemographic profiles, and nor was information collected about existing chronic health conditions other than diabetes. The online questionnaire was not as extensive as the National Health Surveys that are conducted every few years; however, our data are reasonably representative of the population, providing an opportunity to collect ongoing information to monitor the prevalence of modifiable risk factors.

Australian women continue to report low rates of physical activity, fruit and vegetable intake and higher BMI than recommended for good health, despite worldwide health promotion activities aimed at changing these lifestyle factors. Regional prevalence of health risk factors worldwide is associated with the level of human development in each country [52]; therefore, using international guidelines and examples to design country-specific programs that take into account sociocultural differences would be more beneficial to address the particular needs of the people. Further research must be conducted to determine the most effective approaches to regional health promotion, particularly in women. Current programs to support healthy living need to be reviewed and revised to reduce the prevalence of unhealthy lifestyle factors and thus reduce the burden of vascular disease and dementia in women.

### Implications for practice

There is enormous potential to improve the risk profile in women and, in doing so, reduce the number of women and families affected by vascular diseases. Top target areas to address are the low rates of physical activity and low fruit and vegetable intake. Elevated body mass index was the next most significant contributor to risk profile and is also impacted by the previous two lifestyle factors. Because these risk factors are likely to co-occur, specifically designed programs can reduce the combined prevalence. International experience has demonstrated that guidelines and multi-disciplinary programs targeted towards lifestyle change in women were associated with a reduction in deaths, although the presence of risk factors remains suboptimal. Further involvement of allied health clinicians such as dieticians, physiotherapists and psychologists may also reduce the risk. It is important that we prioritise this area in Australia and globally.

## Supporting information

S1 Table Odds ratios–Likelihoods of not meeting different national guidelines based on risk category for other factors. Data are presented as: odds ratio (95% confidence interval). Non-significance is denoted by “NS”. Cells containing increased likelihoods are shaded. The odds ratios represent the likelihood of not meeting national guidelines for row elements if the guidelines for column elements are also not met. For example, the first filled cell in the bottom right indicates that women who did not meet sleep recommendations were 2.6 times more likely to also be at risk for stress.
(DOCX)
