# Correction: Unhealthy habits persist: The ongoing presence of modifiable risk factors for disease in women

**DOI:** 10.1371/journal.pone.0181287

**Published:** 2017-07-07

**Authors:** Cassandra Szoeke, Christa Dang, Philippe Lehert, Martha Hickey, Meg E. Morris, Lorraine Dennerstein, Stephen Campbell

The following information is missing from the Funding section: The National Health and Medical Research Council and Department of Medicine, Faculty of Medicine, Dentistry and Health Sciences funded the researchers who worked on this analysis. The Australian Bureau of Statistics provided population based data used in comparison analysis and web-based data was deidentified and provided by Fleur Streets at Sisu Wellness. Funding for the Healthy Ageing Project (HAP) has been provided by the National Health and Medical Research Council (NHMRC Grants 547600, 1032350 & 1062133), Ramaciotti Foundation, Australian Healthy Ageing Organisation, the Brain Foundation, the Alzheimer's Association (NIA320312), Australian Menopausal Society, Bayer Healthcare, Shepherd Foundation, Scobie and Claire Mackinnon Foundation, Collier Trust Fund, J.O. & J.R. Wicking Trust, Mason Foundation and the Alzheimer's Association of Australia.

## References

[1] Szoeke C, Dang C, Lehert P, Hickey M, Morris ME, Dennerstein L, et al (2017) Unhealthy habits persist: The ongoing presence of modifiable risk factors for disease in women. PLoS ONE 12(4): e0173603 doi:10.1371/journal.pone.0173603 28403144 10.1371/journal.pone.0173603 PMC5389802

