# Supplementary material for: Unhealthy habits persist: The ongoing presence of modifiable risk factors for disease in women
Source: PLoS One. 2017 Apr 12;12(4):e0173603. doi: 10.1371/journal.pone.0173603 (PMC5389802; doi:10.1371/journal.pone.0173603)
Supplement: S1 Table — Data are presented as: odds ratio (95% confidence interval). Non-significance is denoted by “NS”. Cells containing increased likelihoods are shaded. The odds ratios represent the likelihood of not meeting national guidelines for row elements if the guidelines for column elements are also not met. For example, the first filled cell in the bottom right indicates that women who did not meet sleep recommendations were 2.6 times more likely to also be at risk for stress. (DOCX) [file pone.0173603.s001.docx]

|  | *Waist* | *BMI* | *Nutrition* | *Exercise* | *Blood Pressure* | *Cholesterol* | *Glucose* | *Smoking* | *Alcohol* | *Sleep* | *Stress* |
| --- | --- | --- | --- | --- | --- | --- | --- | --- | --- | --- | --- |
| Waist |  | 23.0  (20.92-25.24) | 1.5  (1.43-1.62) | 2.1  (2.01-2.29) | 2.5  (2.08-2.91) | 1.8  (1.66-2.02) | 4.4  (3.66-5.24) | 1.1 NS  (0.97-1.17) | 0.8  (0.77-0.93) | 1.3  (1.22-1.39) | 1.4  (1.28-1.56) |
| BMI | 23.0  (20.92-25.24) |  | 1.4  (1.34-1.48) | 1.9  (1.80-1.99) | 3.0  (2.52-3.53) | 1.6  (1.48-1.78) | 4.4  (3.67-5.38) | 1.1  (1.03-1.20) | 0.7  (0.69-0.81) | 1.3  (1.21-1.34) | 1.3  (1.20-1.34) |
| Nutrition | 1.5  (1.43-1.62) | 1.4  (1.34-1.48) |  | 2.6  (2.51-2.78) | 1.3  (1.13-1.51) | 1.5  (1.35-1.61) | 1.4  (1.21-1.60) | 2.0  (1.87-2.21) | 1.0 NS  (0.89-1.04) | 1.6  (1.52-1.70) | 1.8  (1.67-1.99) |
| Exercise | 2.1  (2.01-2.29) | 1.9  (1.80-1.99) | 2.6  (2.51-2.78) |  | 1.8  (1.55-2.11) | 1.4  (1.26-1.51) | 2.1  (1.82-2.48) | 1.5  (1.37-1.61) | 0.8  (0.70-0.81) | 1.3  (1.24-1.38) | 1.6  (1.51-1.79) |
| Blood Pressure | 2.5  (2.08-2.91) | 3.0  (2.52-3.53) | 1.3  (1.13-1.51) | 1.8  (1.55-2.11) |  | 2.4  (2.06-2.91) | 3.6  (2.88-4.56) | 1.1 NS  (0.88-1.36) | 1.3  (1.03-1.56) | 1.7  (1.48-1.97) | 1.5  (1.19-1.85) |
| Cholesterol | 1.8  (1.66-2.02) | 1.6  (1.48-1.78) | 1.5  (1.35-1.61) | 1.4  (1.26-1.51) | 2.4  (2.06-2.91) |  | 3.2  (2.70-3.73) | 1.1  (1.00-1.32) | 1.0 NS  (0.91-1.21) | 1.4  (1.26-1.51) | 1.5  (1.27-1.67) |
| Glucose | 4.4  (3.66-5.24) | 4.4  (3.67-5.38) | 1.4  (1.21-1.60) | 2.1  (1.82-2.48) | 3.6  (2.88-4.56) | 3.2  (2.70-3.73) |  | 1.0 NS  (0.82-1.27) | 0.8 NS  (0.61-1.01) | 1.6  (1.42-1.88) | 2.0  (1.61-2.42) |
| Smoking | 1.1  (0.97-1.17) | 1.1  (1.03-1.20) | 2.0  (1.87-2.21) | 1.5  (1.37-1.61) | 1.1 NS  (0.88-1.36) | 1.1  (1.00-1.32) | 1.0 NS  (0.82-1.27) |  | 1.9  (1.74-2.12) | 1.5  (1.36-1.59) | 1.3  (1.20-1.50) |
| Alcohol | 0.8  (0.77-0.93) | 0.7  (0.69-0.81) | 1.0 NS  (0.89-1.04) | 0.8  (0.70-0.81) | 1.3  (1.03-1.56) | 1.0 NS  (0.91-1.21) | 0.8 NS  (0.61-1.01) | 1.9  (1.74-2.12) |  | 1.3  (1.20-1.41) | 1.02 NS  (0.91-1.16) |
| Sleep | 1.3  (1.22-1.39) | 1.3  (1.21-1.34) | 1.6  (1.52-1.70) | 1.3  (1.24-1.38) | 1.7  (1.48-1.97) | 1.4  (1.26-1.51) | 1.6  (1.42-1.88) | 1.5  (1.36-1.59) | 1.3  (1.20-1.41) |  | 2.6  (2.41-2.83) |
| Stress | 1.4  (1.28-1.56) | 1.3  (1.20-1.34) | 1.8  (1.67-1.99) | 1.6  (1.51-1.79) | 1.5  (1.19-1.85) | 1.5  (1.27-1.67) | 2.0  (1.61-2.42) | 1.3  (1.20-1.50) | 1.02 NS  (0.91-1.16) | 2.6  (2.41-2.83) |  |
